# Observations on One of the Most Frequent Causes of Abortion

**Published:** 1829-01-01

**Authors:** 


### CLINICAL REVIEW, N° III.

### LXIV.

### MAISON ROYALE DE SANTE.

RECHERCHES SUR UNE DES CAUSES LES PLUS FREQUENTES ET LA MOINS CONNUE DE L'AVORTEMENT, &c. *Researches into one of the most frequent and least understood Causes of Abortion.* By MADAM BOIVIN, M.D. of the University of Marbourg, Chief Superintendant of the MAISON ROYALE DE SANTÉ, &c. &c. Paris, 1828.

The appearance of a work full of dissections and pathological investigations by a LADY, will, no doubt, create some surprise among the *old women* of the profession on this side of the channel! Pathological researches by a lady! Yes! a lady whose anatomical, pathological, and physiological acquirements, as well as her diploma in medicine, need not shrink from comparison with those of a Clarke, a Merriman, a Davis or Davies, a Granville, a Ley, a Ramsbottom, or any of the MAGNATES OBSTETRICI in this proud metropolis. We wonder that Sir Anthony Carlisle did not bring forward MADAM BOIVIN as a practical proof of the soundness of his arguments—for assuredly this lady might fearlessly enter into single combat with the most doughty champion of man-midwifery here. Armed with her shining and terrific PELVIMETER, "Couronné par la Societé Royale," we should like to see Madam B. break a lance or a pair of long forceps with Dr. Davis—or, as the lawyers would say, "join issue" with that celebrated accoucheur, on some of the more intricate problems of mechanical midwifery—as, for example, the "evolution of the fœtus." We are strongly inclined to think that the female M.D. would prove victorious in obstetrical tactics!

Madam Boivin has selected a single (but, as she thinks, a very frequent) cause of abortion, for her investigation, and encumbers not her book with a compilation of what others have written on other causes of this serious accident.

The nature of this cause or class of causes will be best understood by placing before our readers an abstract of a few of the cases detailed in the work before us—cases which are interesting, and rendered less equivocal by *post-mortem* examinations.

*Case* 1. Madame Kall, aged 27, was confined with her first child at the age of 21 years, and delivered by aid of the forceps, after a tedious labour of 60 hours. She became affected with peripneumony, which confined her to her bed for three months afterwards. Subsequently she went through two natural and easy labours. In February, 1826, while coming home from a ball, she caught cold, and had a severe catarrh, which did not, however, prevent her from attending to her domestic concerns for some time. A severe pain becoming fixed in the left side of the chest, twenty leeches were applied, and gave relief. Uterine pains now came on, and a fœtus of five months was expelled. She was carried to the MAISON ROYALE DE SANTÉ, the third day after the abortion, and the fifth day of the peripneumony. In spite of the best medical assistance, she died on the tenth day of the disease.

*Dissection.* There was a large purulent depôt in the right lung, the left being completely tuberculated. There was some inflammation in the abdomen. In the pelvis, the broad ligaments, the fallopian tubes, and the ovaries, were intimately matted and agglutinated to the posterior face of the uterus—the adhesions being so strong that they could not be separated without the aid of the scalpel. In the midst of this mass of adhesions there was a plentiful crop of recent tubercles, from the size of a millet seed to that of a pea.

*Remarks.* The aforesaid pathological condition must have obtained prior to the peripneumony, and must have rendered the enlargement of the uterus a matter of great difficulty. Madam B. thinks, and apparently with reason, that the abortion would have taken place independently of the pulmonic affection. The broad ligaments, in the above condition of disease, and so morbidly adherent to the uterus, could not have stretched, as that organ enlarged in the latter months of uterine gestation—their resistance would excite uterine action—and abortion would be the consequence.*

*Case* 2. M. Delam——, aged 32 years, was brought to the MAISON ROYALE DE SANTÉ, in consequence of a violent hæmorrhage, by which she was reduced to a state of extreme weakness—the pulse not to be felt—the face deadly pale. When a little revived, she gave the following account of herself. She had menstruated at 12, but the returns were not regular, and she was always affected with profuse leuchorrhœa, and obstinate constipation of the bowels. She had been married five years—had received a syphilitic infection from her husband, and underwent the usual treatment—the leuchorrhœa was still more abundant after this complaint, and at 30 she became pregnant, three years after marriage. She was delivered safely at the full term; but aborted in the third month of a second conception, with great loss of blood, which lasted four days, and was the cause of her receptien into the MAISON DE SANTÉ. In addition to the great prostration, the left lower extremity was found to be infiltrated even to the foot—the os tincæ felt larger and harder than natural —and, in endeavouring to raise the uterus on the finger, much resistance was experienced—indeed, it was found impossible to move that organ. The abdomen was distended, and fluctuation was perceptible. Leeches were applied, and various means used—but a slow fever came on, and she died on the 15th day after her entrance into the MAISON.

*Dissection.* Much fluid in the abdomen—liver large—in the mesocolon and sub-peritoneal tissue, tubercles were developed—portions of colon and rectum intimately adherent to the uterus and also to the sacrum—a large purulent depôt in the recto-vaginal fold—fallopian tubes and ovaries agglutinated into a mass and inseparable from the uterus, and when carefully examined, they were found to be disorganized. The substance of the uterus itself was greatly inflamed, and its internal surface sprinkled with black points like petechiæ, whence, no doubt, the prodigious quantity of blood had proceeded. The rectum was taking on a state of disease.

Madam Boivin remarks that there can be no doubt of the swelling and infiltration of the lower extremity being a consequence of the state of disease in the pelvis—a consequence which she has, in numerous instances, seen result from the same. One thing, she observes, is certain—it was impossible for the uterus to

* The cause of those morbid states is attributed by Madam Boivin to neglected bowels, bad air, and extreme irregularity of living.

expand and become developed under such a state of its annexations ;—hence abortion was inevitable.

*Case* 3. Miss L———, aged 24 years, had been subject to obstinate constipation, and leuchorrhœa, especially during the last two years, while residing in Paris. She was carried to the MAISON DE SANTÉ, in consequence of a large loss of blood from the uterus that had succeeded an abortion of three months. The sanguineous discharge had been accompanied by a violent pain in the right loin, hip, and thigh. The pulse was quick, the tongue red. The abdomen gradually enlarged during the next eight days of her residence in the house, but without evident fluctuation. The pain of the lower extremity continued in spite of anodyne frictions. Vomiting, pains in the abdomen, diarrhœa, fever, came on. The state of the uterus was examined, and it was found to be completely immoveable, the os tincæ being gorged. This led Madam B. to believe that there was disease going on in the appendices of the uterus. The fever increased, delirium came on, and she died on the 19th day after her entrance into the house, and on the 22d day from the abortion.

*Dissection.* There was a good deal of peritoneal inflammation, with adhesions among the intestines, especially the small intestines, which were agglutinated in a mass. The colon and rectum were glued to the posterior face of the uterus—the ovaries and fallopian tubes, on both sides, being reduced to a putrid jelly. A portion of cæcum was completely amalgamated by adhesive inflammation, with the fundus of the uterus, in the substance of which was a small white tumour. The internal surface of the organ was livid and gorged with blood.

Madam B. entertains no doubt that disease had been going on long before the abortion, and was the cause, not the consequence, of this event.

It is not necessary to extract any more cases from the large number which the talented authoress has published — some fatal, others not fatal, but with abscesses pointing into the rectum, vagina, and elsewhere, shewing the nature of the disease that produced the abortion. These cases are highly important, and should lead accoucheurs to be attentive to the signs which indicate inflammatory action about the uterus. The poising of the organ on the finger is an ingenious hint of Madam Boivin, and deserves to be remembered.

We are unable to convey any idea of Madame Boivin's pelvimeter, without the aid of plates. The work contains a great number of cases of extra-uterine and false conceptions, with minute dissections, and keen remarks, that deserve the attention of the accoucheur. In short, if we had a plentiful race of Madame Boivins in this country, we should become converts, at once, to the doctrines of Sir Anthony Carlisle.

